# Clinical audit of foot problems in patients with rheumatoid arthritis treated at Counties Manukau District Health Board, Auckland, New Zealand

**DOI:** 10.1186/1757-1146-2-16

**Published:** 2009-05-15

**Authors:** Keith Rome, Peter J Gow, Nicola Dalbeth, Jonathan M Chapman

**Affiliations:** 1 Health and Rehabilitation Research Centre and Discipline of Podiatry, AUT University, Auckland, New Zealand; 2 Department of Rheumatology, Counties Manukau District Health Board, Auckland, New Zealand; 3 Department of Medicine, University of Auckland, Auckland, New Zealand

## Abstract

**Background:**

At diagnosis, 16% of rheumatoid arthritis (RA) patients may have foot joint involvement, increasing to 90% as disease duration increases. This can lead to joint instability, difficulties in walking and limitation in functional ability that restricts activities of daily living. The podiatrist plays an important role in the multidisciplinary team approach to the management of foot problems. The aim of this study was to undertake a clinical audit of foot problems in patients with RA treated at Counties Manukau District Health Board.

**Methods:**

Patients with RA were identified through rheumatological clinics run within CMDHB. 100 patients were eligible for inclusion. Specific foot outcome tools were used to evaluate pain, disability and function. Observation on foot lesions were noted and previous history of foot assessment, footwear/insoles and foot surgery were evaluated.

**Results:**

The median age of the cohort was 60 (IQR: 51–64) years old with median disease duration of 15 (IQR: 7.3–25) years. Over 85% presented with foot lesions that included corns and callus over the forefoot region and lesser toe deformities. Moderate to high disability was noted. High levels of forefoot structural damage were observed. 76% had not seen a podiatrist and 77% reported no previous formal foot assessment. 40% had been seen at the orthotic centre for specialised footwear and insoles. 27% of RA patients reported previous foot surgery. A large proportion of patients wore inappropriate footwear.

**Conclusion:**

This clinical audit suggests that the majority of RA patients suffer from foot problems. Future recommendations include the provision of a podiatrist within the current CMDHB multidisciplinary rheumatology team to ensure better services for RA patients with foot problems.

## Introduction

Rheumatoid arthritis (RA) is the most common type of inflammatory arthritis [1]. The prevalence rate of RA in New Zealand has been reported to be between 0.79–2.6% [2,3]. When untreated, the disease can progress rapidly, causing swelling and damage to cartilage and bone around the joints. Any joint may be affected, but hands, wrists and feet are most often involved [4].

The main symptoms of RA are severe pain, stiffness, fatigue and loss of mobility. 42% of RA patients are registered disabled within 3 yrs of diagnosis [2]. 80% are moderately to severely disabled within 20 yrs. At diagnosis, 16% of RA patients may have foot joint involvement [5] increasing to 90% as disease duration increases [5,6]. This can lead to joint instability, difficulties in walking and limitation in functional ability that restricts activities of daily living [6]. The talo-navicular joint is the most commonly affected; subtalar joint involvement shows a similar pattern, with an increase of 25% between 5–10 years of duration [7]. Deformity of the tarsal joints and forefoot also occurs with disease progression [8].

Williams and Bowden [9] reported that the evidence base for dedicated podiatry as part of multidisciplinary foot clinics in diabetes is well established, but that this has yet to be achieved for rheumatology services [10]. However, the role of the podiatrist in the rheumatology team is becoming recognised as a vital component in the integrated care given to patients by the multidisciplinary team [11,12]. Increasingly consultants and their teams are requesting specialist foot care services and it is suggested that the podiatrist is a key practitioner in the management of patients with musculoskeletal disease [11-13]. It has been recommended that patients should understand the role and have access to a podiatrist [9]. The podiatrist's role is to identify, diagnose and treat disorders, diseases and deformities of the feet and legs and implement appropriate and timely care. Additionally, podiatrists also monitor foot health status, provide education and support in enabling behaviour change in lifestyle, and may be the only health-care professional that the patient sees on a regular basis. Therefore, they may arguably act as gatekeepers to other members of the multi-disciplinary team. This may be provided directly by a podiatrist or in association with other healthcare team members as required by the individual's foot problems [4].

The goal of the podiatry element of rheumatology care is to reduce foot-related pain, maintain/improve foot function and thus mobility, while protecting skin and other tissues from damage [4]

Despite this recognition, it is generally perceived that access to podiatry services for patients with rheumatic diseases appears to be varied and in some instances absent, especially in New Zealand. Podiatrists have a prominent role to play in symptom relief and improving quality of life because involvement of the feet, even to a mild degree, is a significant marker for impaired mobility, functional incapacity and negative psychosocial impact. Foot pathology contributes to difficulty with walking in about 75% of people with RA, and is the main or only cause of walking difficulty in 25% [1]. In the foot, joint pain and stiffness is the most common initial presentation, but a range of other features, including tenosynovitis, nodule formation and tarsal tunnel syndrome may also present, reflecting widespread soft-tissue involvement [13,14].

Based upon the Arthritis and Musculoskeletal Alliance (ARMA) Standards of Care for People with Foot Health Problems and Inflammatory Arthritis [15] the purpose of this audit is to provide a benchmark by which podiatric service standards may be evaluated by all stakeholders. Therefore, the aim of this study is to identify the nature of foot problems experienced by patients attending rheumatology outpatient clinics at Counties Manukau District Health Board (CMDHB) and to ascertain the availability of podiatric services for these patients.

## Method

### Patients

The clinical audit was conducted over 12 weeks between December 2008 and March 2009. Sample size was determined by a fixed recruitment period for this clinical audit.

Ethical approval was obtained from the Northern Regional Ethics Committee. All patients received information regarding the study. A convenience sample of 100 RA patients was recruited from rheumatology outpatient services based at CMDHB, Auckland, New Zealand. One examiner (JC) interviewed and assessed all patients. Patients were eligible if they had a confirmed diagnosis of RA (satisfying the 1987 American Rheumatism Association revised criteria [16].

### Demographic characteristics

Age, ethnicity, gender, occupation, disease duration, Health Assessment Questionnaire (HAQ) [17] and current pharmacological management that includes non-steroidal anti-inflammatory drugs (NSAIDs), methotrexate, other disease modifying anti-rheumatic drugs (DMARDs), prednisone and biologic therapies were recorded for each patient. Blood results that included erythrocyte sedimentation rate (ESR), C-reactive protein (CRP) and the presence of radiographic erosions were recorded from the patient's case notes.

### Foot and ankle assessment

Foot and ankle assessment were based upon the recommendations from the Standards of Care for People with Musculoskeletal Foot Health Problems [16]. Assessments included:

(i) Measures of structure and function;

(ii) Lifestyle and social factors;

(iii) Footwear suitability;

(iv) Tissue viability and skin and nail assessments;

(v) Baseline measures of foot impairment;

(vi) The impact of any previous interventions, including surgery and foot orthoses.

The musculoskeletal foot examination included documentation of foot and toe deformities, foot type, joint swelling, pain and instability, plantar callus and foot ulceration. Baseline measures of pain, function and health status were also assessed [12,17].

### Procedure

Fore- and rearfoot deformities were quantified using the Structural Index score [18], which considers hallux valgus, metatarsal phalangeal (MTP) subluxation, 5^th ^MTP exostosis, and claw/hammer toe deformities for the forefoot (range 0–12) and calcaneus valgus/varus angle, ankle range of motion and pes planus/cavus deformities for the rearfoot (range 0–7). Lesser toe deformities (hammer, mallet, and claw toes) were scored one point each, as were hyperkeratotic lesions (corns and calluses). Other abnormal bony prominences, such as Tailor's bunions and exostoses, were also scored one point [19]. Patients were asked if they received professional foot care and what interventions were used (such as palliative care, foot orthoses or specialist footwear). Similarly those patients who had been provided with foot orthoses by the local orthotic centre were asked about the suitability of the devices and if they had been beneficial in reducing foot pain. Finally, the assessing podiatrist asked if the patients' had undergone previous foot surgery.

### Disease measurement

Disease impact was measured using the Leeds Foot Impact Scale [12]. This self completed questionnaire comprises two subscales for impairment/footwear (LFISIF) and activity limitation/participation restriction (LFISAP). The former contains 21 items related to foot pain and joint stiffness as well as footwear related impairments and the latter contains 30 items related to activity limitation and participation restriction [12].

### Footwear characteristics

An objective assessment of footwear was carried out by the podiatrist, to ascertain the type and appropriateness of the participant's footwear. The assessment included shoe style: selected from a list of 16 basic shoe styles and included the terms sandals, mules and jandals [20]. Sandals are defined as shoes consisting of a sole fastened to the foot by thongs or straps. A mule shoe is a type of shoe that is backless and often closed-toed. The term jandals, used predominantly in New Zealand and the South Pacific (also known as flip-flops in the UK and US and thongs in Australia) are flat, backless, usually rubber sandal consisting of a flat sole held loosely on the foot by a Y-shaped strap that passes between the first and second toes and around either side of the foot.

### Data analysis

Data were analysed using SPSS 15.0 for Windows. Pharmacological management, gender, occupation, ethnicity and general footwear scores were described as percentages. All other demographic characteristics were described as the median (interquartile range – IQR).

## Results

### Participant demographics and disease characteristics

One hundred (100) RA patients were recruited into the study with a median age of 60 (IQR: 51–64) years old with 79% being women. The results demonstrated a ratio of 4:1 female: males (Table 1). Fifty-seven patients (57%) were Caucasian with 14% being Maori and 14% Pacific Islanders. The median duration of disease was 15 (IQR: 7.3–25) years which suggests a well-established disease with levels of functional disability. In spite of the general prevalence of moderate disease activity, 39% of patients were employed.

**Table 1:** Demographic & clinical characteristics

Demographic characteristics	Value
Median (IQR) Age (years)	60 (51–64)

% Gender (F:M)	4:1 (79% females, 21% males)

% Ethnicity	Caucasian: 57%,
	Pacific Island 14%,
	Maori: 14%,
	Asian: 11%,
	Non-European Caucasian: 4%

Median (IQR) disease duration (years)	15 (7.3–25)

Working: (%)	34%

Not working/Beneficiary: (%)	15%

Retired: (%)	31%

Housewife/homemaker: (%)	15%

**Clinical characteristics**	

Median (IQR) HAQ Score (0–3)	0.9 (0.4, 1.3)

% radiographic erosions	Yes: 67%; No: 33%

**Pharmacological management**	

NSAIDS: n (%)	62 (64%)

Methotrexate: n (%)	65 (67%)

Other DMARDS: n (%)	41 (42%)

Prednisone: n (%)	32 (33%)

Biologics: n (%)	17 (17%)

**Blood investigations**	

Median (IQR) ESR (mg/hr)	27.0 (12.0; 42.3)

Median (IQR) CRP (mm/L)	7.9 (3.8; 7.3)

Sixteen percent (16%) of patients also had diabetes Sixty-seven percent (67%) of patients presented with erosions on X-rays. Sixty-four percent (64%) of patients were treated with NSAIDs, 67% specifically with methotrexate, 42% with other DMARDs, 33% with prednisone and 17% with biologic therapies.

### Foot impairments

Patients in the current study had high-to severe (LFISIF >7 point, LFISAP >10 points) levels of foot impairment and disability on the LFIS subscales (Table 2). The forefoot structural index demonstrated severe structural problems but the rearfoot structural indices demonstrated moderate problems. Over two-thirds of patients were observed with hallux valgus (bunions). Over 85% of patients in the study presented with symptomatic callus under the plantar surface of the foot and/or on the toes.

**Table 2:** Foot & ankle characteristics

Foot characteristics	Score
Median (IQR) Forefoot Structural Index (range 0–12)	9 (4–14)

Median (IQR) Rearfoot Structural Index (range 0–7)	2 (2–4)

Median (IQR) Leeds Foot Impact Scale impairment/footwear (range 0–21)	11 (8–14)

Median (IQR) Leeds Foot Impact Scale activity limitation/participation restriction (range 22–51)	18 (11–23)

Total Median (IQR) Leeds Foot Impact Scale	29 (20–36)

Median (IQR) Foot Problem Score	7 (5–13)

Hallux Valgus: (%)	64%

Number and Frequency of Foot Lesions: n (%)	99 (79%)

Forefoot Callus: (%)	63%

Lesser Toe Deformities: (%)	86%

Previous Foot Assessment: n (%)	Yes: (23%) No: (77%)

Previous Podiatry: n (%)	Yes: (24%) No: (76%)

Previous Foot Surgery: n (%)	Yes: (27%) No: (73%)

Previous Orthotics: n (%)	Yes: (40%) No: (60%)

### Podiatric care

Concerning podiatric intervention, 24% of RA patients reported they have been seen by a podiatrist and over 40% of patients used the orthotic centre. Because of the absence of a podiatry service at CMDHB for patients other than those with diabetes, no participant in the current study had received podiatric intervention by a qualified podiatrist specialising in the management of rheumatic diseases. 27% of patients reported having foot surgery (Table 2). The majority of RA patients wore open-type footwear such as sandals (21%), jandals (10%) or mules (10%). Only nine patients wore surgical footwear (Table 3).

**Table 3:** General footwear assessment

General footwear type	%
Sandal	21%

Mule	13%

Jandals	10%

Walking Shoe	10%

Athletic Shoe	9%

Moccasin	9%

Surgical/Orthotic Shoe	9%

Boot	6%

High Heel	4%

Backless Slipper	2%

Court Shoe	2%

Oxford Shoe	2%

Socks only	1%

Slipper	1%

Unable to assess footwear	1%

## Discussion

The purpose of this study was to undertake a clinical audit evaluating current RA foot care services in Counties Manukau. Overall, this study demonstrates that in this particular outpatient clinic, poor foot health and foot pain is highly prevalent in patients with rheumatic diseases. Over 85% of patients with RA had foot involvement ranging from callus, corns and lesser toe deformities. The study also demonstrated moderate impairment and limited activity using the Leeds Foot Impact Scale [12] suggesting the majority of patients suffer with long-term disability from this chronic condition.

The problem of footwear was highlighted in the audit. The majority of patients were observed wearing footwear that could be described as inappropriate for those patients with severe pain and disability and included sandals, mules and jandals. The lack of support mechanisms, cushioning and protection of toe regions may contribute to pain and disability.

The current clinical audit demonstrated 16% of RA patients presented with diabetes suggesting that patients with autoimmune disorders, and/or taking medication that compromises the immune system should be considered at risk of infection and foot ulceration, due to a lack of protection, especially on the plantar surface of the feet. Likewise, patients with micro-vascular and/or large vessel disease and foot deformity are also at risk of foot trauma, ulceration and subsequent infection [9]. The use of podiatrists and management programmes that includes advice of foot health education, appropriate footwear and prescription of foot orthoses is essential if we are to reduce the impact of foot problems in this patient group.

The results from the current audit are similar to UK audits [3,9]. Recent reports from the UK recommend the need for more consistent provision of specialist care for patients with RA and better implementation of guidance and best practice [4,21]. However, there is no similar data or recommendations for New Zealand, and there are no previous studies of foot problems in New Zealand patients with rheumatic diseases.

The results of this study support the case for a specialist podiatrist to manage patients with rheumatic diseases in this locality, whatever the patient's age or stage of disease. This audit recommends that in order to identify patients with foot problems, their consultant or specialist nurse should question patients about their feet. If foot problems are identified, a referral to the specialist podiatrist should be made. Patients with disabling foot pain, or who are at risk of foot ulceration, should receive priority foot care [9,22]. Foot orthoses should be considered for patients recently diagnosed with RA as this intervention has been demonstrated to reduce pain and the effects of abnormal joint function in the foot [23].

The current audit demonstrated that RA patients reported podiatry was "very useful". However, patients perception of the term podiatry was related only to toenail cutting, and corn reduction rather than for structural modifications. The role of the podiatrist within rheumatology involves a range of different assessments and interventions [4]. An assessment of the lower limbs may include the skin, vascular and neurological systems, the musculoskeletal structures and walking. Specialist prescription footwear should also be available for patients who cannot fit into appropriate retail footwear and in this domain; both podiatrists and orthotists should collaborate to achieve the optimal clinical outcome [9]. Working with surgeons, in relation to appropriateness for foot surgery, should also be considered, as this option is often overlooked by podiatrists. This study demonstrates that there is an unmet need for specialist podiatry in patients attending this particular rheumatology outpatient clinic. A mechanism should be in place whereby everyone with a diagnosis of inflammatory arthritis receives a foot examination within three months of diagnosis [16].

Patients with RA should be provided with information and education to enable them to recognise the signs of these variations and understand what to do if variations occur. Increased systemic disease activity can accelerate changes in foot pathology so consideration must be taken of local as well as systemic factors. A recent study undertaken in the UK using a self-management foot care programme for 30 patients with RA demonstrated that just over 50% of patients were physically able to undertake some aspects of self-managed foot care, including nail clipping and filing, callus filing and daily hygiene and inspection [13]. A clinical audit of 139 rheumatoid patients undertaken in the UK reported that poor foot health and foot pain as being common in patients with rheumatic diseases [9]. The authors highlighted that the lack of foot care could lead to reduction in mobility and in some cases serious complications and recommended that a specialist and dedicated foot care service needs to be provided for these patients [9].

## Conclusion

The current study has highlighted patients with RA have an increased need for a range of basic foot care services. There is evidence from the current literature that early intervention for existing or potential foot problems can improve long-term outcomes. Baseline foot examination can identify people with existing or imminent needs and provide a comparator for assessment. Regular assessments that document the rate of structural change can aid treatment decisions and improves outcomes. An annual musculoskeletal, vascular and neurological assessment, which includes an assessment of the lower limbs and feet, will help identify problems early. Future developments may incorporate self-educational programmes and the need for podiatrists to be part of the rheumatological multidisciplinary team. Overall, this study showed that opportunities for innovation and improvement in RA services exist and need to be pursued vigorously including the development of a business case for a combined DHB-academic post in podiatry.

## Competing interests

The authors declare that they have no competing interests.

## Authors' contributions

KR, PG and ND conceived and designed the study. JC collected and inputted the data. KR, PG and ND conducted the statistical analysis. KR and JC compiled the data and drafted the manuscript and ND and PG contributed to the drafting of the manuscript. All authors read and approved the final manuscript.
